# Amlodipine limits microglia activation and cognitive dysfunction in aged hypertensive mice

**DOI:** 10.1097/HJH.0000000000003445

**Published:** 2023-04-05

**Authors:** Danielle Kerkhofs, Robin Helgers, Denise Hermes, Hellen P.J. Steinbusch, Helma Van Essen, Peter Leenders, Jos Prickaerts, Julie Staals, Erik A. Biessen, Robert J. Van Oostenbrugge, Sébastien Foulquier

**Affiliations:** a Department of Neurology, Maastricht University Medical Center; b Department of Pathology; c CARIM, School for Cardiovascular Diseases; d Department of Psychiatry and Neuropsychology; e MH&NS, School for Mental Health and Neurosciences, Maastricht University, Maastricht, The Netherlands; f IMCAR, Institute for Molecular Cardiology Research, RWTH Aachen, Aachen, Germany; g Department of Pharmacology-Toxicology, Maastricht University, Maastricht, The Netherlands

**Keywords:** calcium channel blockers, cerebral small vessel diseases, cognitive dysfunction, microglia, neuroinflammation

## Abstract

**Background:**

SBP and blood pressure variability are independent risk factors for cerebral small vessel disease, a leading cause for stroke and dementia. Calcium-channel blockers are known to reduce blood pressure variability and may thus offer benefit against dementia. Beyond this effect, the impact of calcium-channel blockers on hypertension-induced neuroinflammation, and especially, microglial phenotype remains unknown. We aimed to study the ability of amlopidine to alleviate microglia inflammation, and slow down cognitive dysfunction in aged hypertensive mice.

**Methods:**

Hypertensive BPH/2J and normotensive BPN/3J mice were studied until 12 months of age. Hypertensive mice were untreated or received amlodipine (10 mg/kg per day). Blood pressure parameters were measured by telemetry and tail cuff plethysmography. Mice underwent repeated series of cognitive tasks. Brain immunohistochemistry was performed to study blood–brain barrier dysfunction and microglial pro-inflammatory phenotype (CD68^+^Iba1^+^ cells; morphological analysis).

**Results:**

Amlodipine normalized SBP over the entire life span and decreased blood pressure variability. BPH/2J mice exhibited impaired short-term memory that was prevented by amlodipine at 12 months (discrimination index 0.41 ± 0.25 in amlodipine-treated vs. 0.14 ± 0.15 in untreated BPH/2J mice, *P* = 0.02). Amlopidine treatment of BPH/2J did not prevent blood–brain barrier leakage, a measure of cerebral small vessel disease, but limited its size. Microglia's inflammatory phenotype in BPH/2J, characterized by an increased number of Iba1^+^CD68^+^ cells, increased soma size and shortened processes, was partly reduced by amlodipine.

**Conclusion:**

Amlodipine attenuated the short-term memory impairment in aged hypertensive mice. Beyond its blood pressure lowering capacity, amlodipine may be cerebroprotective by modulating neuroinflammation.

## INTRODUCTION

Cerebral small vessel disease (cSVD) encompasses all diseases that affect the brain small arteries, arterioles and capillaries [1]. The most common type of cSVD is an age and hypertension-related vasculopathy, which is the main cause of vascular cognitive impairment (VCI), vascular dementia and lacunar stroke [2]. The understanding of the exact contribution of hypertension to pathobiological mechanisms of cSVD is still limited and needs to be unravelled for future therapeutic and preventive options. The main mechanistic insights in hypertension-induced cSVD have been gained using young adult animals in which hypertension was induced artificially. In the present study, we used aged spontaneous hypertensive animals. The BPH/2J mice with a genetic basis for spontaneous lifelong hypertension is an inbred strain developed in the 1970 s alongside a normotensive strain (BPN/3J) [3]. The BPH/2J mice manifest some important features of essential hypertension, including cardiac hypertrophy, neurohumoral dysfunction and sympathetic activation [4,5].

An increased permeability of the blood–brain barrier (BBB) seems to play an important role in the link between hypertension and structural brain abnormalities, such as white matter damage, which develop in the course of cSVD [6–11]. The BBB is a part of the neurovascular unit (NVU), which is formed by endothelial cells interconnected through tight junctions and surrounded by pericytes, astrocytes and microglia cells [12]. The main function of this NVU is the maintenance of brain homeostasis by regulating the exchange of nutrients, oxygen and waste products between the blood stream and brain parenchyma [13,14]. Increased permeability of the BBB can consequently lead to the leakage of plasma components into the brain parenchyma, which in turn can lead to an activation of inflammatory cells of the NVU [15,16].

Whether antihypertensive drugs, beyond their BP-lowering effects, could have an impact on BBB permeability and neuroinflammation is unknown. Among the different antihypertensive drug classes that have been assessed for stroke and dementia prevention, calcium-channel blockers (CCBs) have shown to offer greater benefit than other antihypertensive classes [17–19]. CCBs reduce blood pressure variability (BPv) in addition to BP lowering [20,21] and BPv is another important independent risk factor for cSVD [22–25]. Furthermore, it has been shown that CCBs are able to reduce microglial activation *in vitro*, but this has not been studied *in vivo*[26].

In this study, we aimed to investigate the effect of the CCB amlodipine on BP, BPv, cognitive function, BBB integrity and neuroinflammation in aged hypertensive mice. We hypothesized that amlodipine would normalize BP and BPv and limit neuroinflammation thereby reducing cognitive decline over time.

## MATERIALS AND METHODS

### Animals

Two to three-months-old hypertensive BPH/2J (BPH, Jackson Lab 003005) and normotensive BPN/3J (BPN, Jackson Lab 003004) mice were allocated to three study groups: untreated BPH/2J (BPH) untreated BPN/3J (BPN) and BPH/2J treated with the calcium channel blocker amlodipine (BPH + A). A first series of mice (*n* = 3–4 per group) was implanted with blood pressure telemetry transmitters for the characterization of blood pressure until 9 months of age. Cognitive function, SBP and histological parameters were assessed until 12 months of age in a second series of mice (*n* = 9–11 per group). Amlodipine (PHR1185; Merck KGaA, Darmstadt, Germany) was provided in the drinking water of the mice (10 mg/kg/day). Mice were exposed to a normal 12-h day-night cycle, with ad libitum access to water and food. Body weight of the animals was measured weekly. All animal experiments were approved by the regulatory authority of Maastricht University and were performed at the Maastricht University in compliance with the national and European guidelines (AVD1070020173666).

### Blood pressure measurements

In the first series of mice, radiotelemetry transmitters (DSI PhysioTel PA-C10 Transmitter; Data Sciences International, Saint Paul, Minnesota, USA) were implanted under isoflurane anaesthesia with the catheter inserted in the carotid artery and the transmitter along the right flank. Mice were housed individually after implantation and there was at least a 10-day recovery until the first measurement. Every month, systolic arterial pressure, diastolic arterial pressure and heart rate were continuously measured for 48 h with a sampling rate at 500 Hz. The software package Ponemah 6.41 (DSI) was used for recording and analysis. As a metric for systolic BPv, the coefficient of variation was calculated over a fixed period of 24 h of the measurement: CV = standard deviation (SD) SBP / mean SBP x 100%.

In the second series of mice, SBP was measured by tail-cuff plethysmography (CODA, Kent Scientific) every 2–3 months during the study period [27]. The mice were placed in a restrainer on a warming platform. Before the start of the measurements, the mice were allowed to acclimate for 15 min. Each recorded session consisted of 30 accurate inflation-deflation cycles.

### Cognitive testing

Short-term memory and working memory were measured with the object location task (OLT) and Y-maze alternation task, respectively, at month 4, 8 and 12. Both OLT and novel object recognition task (NORT) are common behavioural tasks that can be used to assess short-term and long-term memory in rodents (depending on the inter-trial period). The OLT has been described to rely mainly on hippocampal function, while the NORT involves multiple brain areas. As such, some studies have found that the NORT is not always suited to identify hippocampal lesions [27,28]. In the present study, we have therefore chosen to use the OLT over the NORT to assess short-term memory in mice. The spatial OLT consisted of a learning trial (T1) and a test trial (T2) with a 1 h interval in between [16]. During T1, mice were allowed to explore freely for 4 min the circular arena where a set of two identical objects were placed symmetrically in the centre. Next, the mice spent 1 h in their home cage and were then again placed in the arena for 4 min free exploration. In this T2, one of the objects had been moved to a new location, while all other stimuli remained the same as in the first trial. If mice remember the previous location of the objects, they will naturally spend more time exploring the newly located object. An investigator blinded to the experimental groups manually scored the time that the mice spent exploring each object. Although the investigator was blinded for the treated and untreated BPH mouse groups, it was not possible to keep the investigator blinded for the BPN mouse group due to difference in coat colour between BPH and BPN mice. Trials were excluded from the analysis when the total exploration time was too low to result in reliable exploration times, that is 6 s. The difference between object exploration times in T2 divided by the total exploration time resulted in the discrimination index d2. Functional spatial short-term memory is reflected by a d2 index higher than chance level, that is zero, meaning that the newly located object has been explored for a longer time than the object that had not been relocated. The OLT was performed on 2 days, with a 1 day interval in between, whereafter the d2 index was averaged for each mouse.

The Y-maze spontaneous alternation task was used to assess spatial working memory [29]. At the start, each mouse was placed in a randomly selected arm of the Y-maze and was allowed to freely explore the maze for 6 min. The number of the different arm entries was recorded using EthoVision (Noldus, Wageningen, The Netherlands). Every ‘triad’, meaning a successful alternation of all three arms of the maze, was counted for each mouse and the alternation percentage was calculated as the number of triads divided by the maximum possible number of alternations per mouse. Indicative for a functional working memory is an alternation rate above 50%, that is the chance-level probability of choosing the unfamiliar arm.

Long-term spatial memory was examined using the Barnes maze test [30]. The Barnes maze consists of a circular arena with a diameter of 950 mm. There were 12 circular holes (50 mm in diameter) equally spaced around the perimeter. Only one hole will give access to escaping the maze via an escape box and the mouse is then directly placed back into its home cage. Trials were recorded and animals tracked using EthoVision (Noldus Equipment, Wageningen, the Netherlands). For each trial, the mouse was placed into the start box, which was put in the centre of the arena. The trial ended when the mouse entered the escape hole or when 3 min had elapsed (the mouse was then directed by the investigator to the escape hole). Between trials, the maze floor was cleaned with 10% ethanol in water to prevent olfactory cues. The mice received four trials per day and were trained on four consecutive days. The distance (cm) travelled before entering the escape hole was recorded for assessing the learning curve. At day 5, a probe trial was conducted in which the escape hole was closed, and the mouse was allowed to explore the arena for 2 min. The time spent in the zone surrounding the closed escape hole (target quadrant) was measured.

### Tissue collection

Animals were sacrificed at the age of 12 months. Before sacrifice, mice were anesthetized with isoflurane and injected intravenously with 70 kDa-dextran Texas Red (100 μl; 2.5 mg/ml in sterile NaCl 0.9%, 15 min circulation time; D1864, Thermo Fisher) that was used to detect the presence of BBB leaks by histology. Brains were postfixed with 4% paraformaldehyde overnight at 4°C and then transferred into Tris-buffered saline (TBS) with 1% sodium azide at 4°C for preservation. Fifty micrometre thick coronal sections were prepared using a vibratome (VT1200S; Leica Biosystems, Nussloch GmbH, Germany).

### Brain immunohistochemistry

Free-floating coronal sections were washed with TBS, permeabilized with 0.5% Triton in TBS and blocked with 1% bovine serum albumin under shaking condition at room temperature for 2 h. Sections were incubated with primary antibodies anti-Iba1 (rabbit polyclonal; C-terminus Iba1; 1 : 1000; Wako 019–19741), anti-CD68 (rat monoclonal, macrosialin protein; 1 : 500, Bio-Rad MCA1957) or antimouse IgG Dy light 405 (donkey polyclonal, IgG (H+L); 1 : 200, Jackson ImmunoResearch N715–475–150) overnight at 4°C. After washing, the sections were incubated with secondary antibodies including donkey antirabbit Alexa Fluor 488 [donkey polyclonal, IgG (H+L); 1 : 500, Thermo Fisher A21206, 2 h at RT] or donkey antirat biotin (Biotin-SP AffiniPure, donkey polyclonal, IgG (H+L); 1 : 400, Jackson Immunoresearch 712–065–150, 2 h at RT), followed by conjugation with streptavidin Alexa Fluor 647 (1 : 500, Thermo Fisher S32357, 1.5 h at RT). Brain sections were mounted on gelatin-coated microscopic slides using fluorescence mounting medium (Dako S3023).

### Image acquisition and analysis

Brain sections were imaged with a confocal microscope (Leica DMI 4000). Identification of BBB leakages and image analysis were performed by a blinded investigator using ImageJ (Fiji Distribution, NIH). BBB leakages were identified as dextran and/or IgG signals with an intense core and diffuse borders. A series of six slices per brain were screened to identify and localize leakages. Leakage size was assessed by determining the level of plasmatic IgG proteins into the brain parenchyma as done previously [16]. Maximally projected images (x = 175; y = 175; z = 20 μm) were used to assess the IgG leakage area in mm^2^. The number of Iba-1^+^ and Iba-1^+^ CD68^+^ cells were manually counted per leakage. Microglia density was also assessed in areas without BBB leakages: Iba1^+^ and CD68^+^ Iba1^+^cells cells were counted on eight randomly selected volumes within the cortex and the striatum (x = 276; y = 276; z = 20 μm; *n* = 8). Microglia morphology, including cell soma size and length of microglia ramifications, was assessed using Iba1 signal and WIS-NeuroMath software as done previously [16,31] using the following parameters: noise level, 15; measure type, cell morphology; segmentation type, threshold; minimal cell intensity, 90; minimal area, 20; maximal area, 1532; minimal diameter, 4; maximal axial ratio, 8; minimal neurite length, 5.

### Statistical analyses

All statistical analyses were performed with GraphPad Prism 9 software (GraphPad Software, LLC, Boston, Massachusetts, USA). Data are expressed as mean ± standard error of the mean (SEM). One-way and two-way analyses of variance (ANOVA) were followed by Tuckey and Bonferroni multiple comparison posthoc tests as indicated in the corresponding figures. A *P* value less than 0.05 was considered as statistically significant.

## RESULTS

### Amlodipine normalizes blood pressure over the entire life span

**FIGURE 1:**
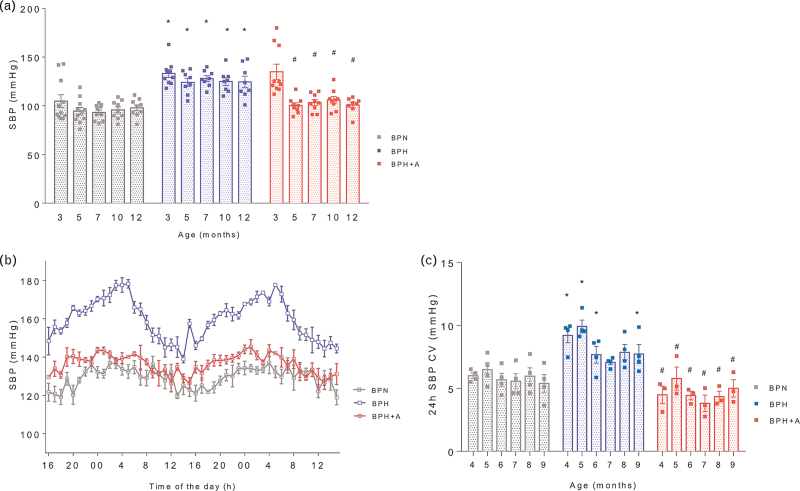
Amlodipine reduces SBP and 24-h blood pressure variability. (a) SBP values measured by tail-cuff plethysmography from 3 to 12 months of age (2-W ANOVA *P*_int_ = 0.03; *P*_time_ < 0.001; *P*_group_ < 0.0001; Bonferroni's post hoc test: ^∗^*P* < 0.01 vs. BPN; ^∗∗^*P* < 0.01 vs. BPH; *n* = 9–11 per group. (b) Forty-eight hour SBP telemetry measurement at 8 months of age (*n* = 3–4 per group). (c) Twenty-four hour telemetry SBP variability expressed as the coefficient of variation (CV) at different time points over the study period (2-W ANOVA *P*_int_ > 0.05; *P*_time_ < 0.05; *P*_group_ < 0.001; Tukey's multiple comparison test: ^∗^*P* < 0.05 vs. BPN; ^∗∗^*P* < 0.05 vs. BPH). CV, coefficient of variation.

SBP was higher in BPH vs. BPN at 3 months of age (133 ± 13 vs. 105 ± 22 mmHg, *P* < 0.01) and this persisted over the whole study period (Fig. 1a, *P* < 0.01 vs. BPN). Amlodipine treatment was initiated in BPH+A group after the first measurement at 3 months, and it reduced SBP at 5 months of age and at the later study timepoints (Fig. 1a, *P* < 0.01 vs. BPH). Forty-eight hour telemetry measurements confirmed the increased SBP in BPH mice and the normalization after amlodipine treatment (Fig. 1c). SBP variability, expressed as coefficient of variation, was higher in BPH mice vs. BPN mice (Fig. 1c, *P* < 0.05 vs. BPN) and was reduced by amlodipine (Fig. 1c, *P* < 0.05 vs. BPH).

### Amlodipine attenuates short-term memory impairment in aged hypertensive mice

**FIGURE 2:**
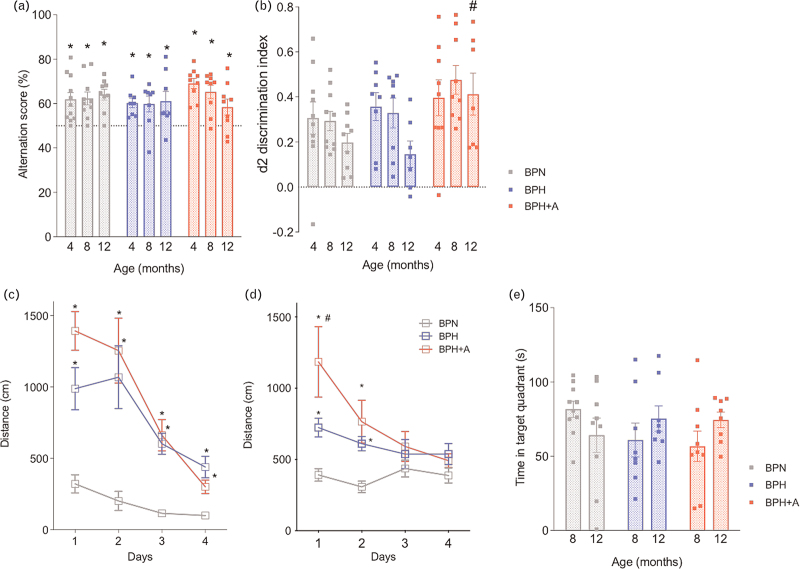
Impact of hypertension and amlodipine treatment on cognitive function. (a) Spatial working memory was unaltered in BPH mice in the y-maze alternation task (*t*-test: ^∗^*P* < 0.05 vs. 50%; *n* = 8–10 per group). **(**b**)** The decrease in spatial short-term memory (d2 discrimination index) in BPH in the Object Location Task was prevented by amlodipine (2-W ANOVA *P*_int_ > 0.05; *P*_time_ = 0.072; *P*_group_ < 0.01; Tukey's multiple comparison test: ^∗∗^*P* < 0.05 vs. BPH; *n* = 8–10 per group). (c--e) Spatial learning and long-term memory were assessed using the Barnes Maze Task. **(**c**)** At 8 months of age, the distance to escape was higher in BPH and BPH+A vs. BPN (2-W ANOVA *P*_int_ < 0.005; *P*_time_ < 0.001; *P*_group_ < 0.001; Tukey's multiple comparison test: ^∗^*P* < 0.05. vs. BPN; *n* = 8–10 per group). (d) At 12 months of age, the distance to escape was higher in BPH and BPH+A vs. BPN on the first 2 days and the distance to escape was higher in BPH+A on day 1 vs. BPH (2-W ANOVA *P*_int_ < 0.005; *P*_time_ < 0.005; *P*_group_ < 0.005; Tukey's multiple comparison test: ^∗^*P* < 0.05 vs. BPN; ^∗∗^*P* < 0.05 vs. BPH; *n* = 8–10 per group). **(**e**)** Long-term spatial memory as assessed by the time spent in the target quadrant during the probe trial, did not differ between groups (2-W ANOVA *P*_int_ > 0.05; *P*_time_ > 0.05; *P*_group_ > 0.05; *n* = 8–10 per group).

Mice were first tested on the y-maze spontaneous alternation task to assess spatial working memory. The alternation rate was significantly above the chance level in all the study groups (Fig. 2a). Short-term memory was measured with the OLT. In young (4 months) and middle-aged mice (8 months), there was no difference in the discrimination index between the study groups. In aged groups (12 months), the discrimination index decreased in both BPH and BPN mice, and furthermore, BPH mice had a significantly decreased discrimination index compared with BPH+A mice (Fig. 2b). Long-term spatial memory was examined using the Barnes maze test. All mice groups showed spatial learning over the 4-day test, as indicated by the reduction in distance travelled to reach the escape hole (Fig. 2c, d). At 8 months of age, both hypertensive mouse groups, BPH and BPH+A, required a significantly longer distance to reach the escape hole compared to BPN mice (Fig. 2c). This difference was maintained at 12 months of age but only on day 1 and 2, while there was no difference anymore on day 3 and 4 for the escape distance between the groups (Fig. 2d). In addition, spatial learning was worst in BPH+A compared with BPH mice but only at day 1 as shown by the longer escape distance. For the probe trial at day 5, there was no difference between groups in the time spent in the target quadrant (Fig. 2e).

### Amlodipine and blood–brain leakages

**FIGURE 3:**
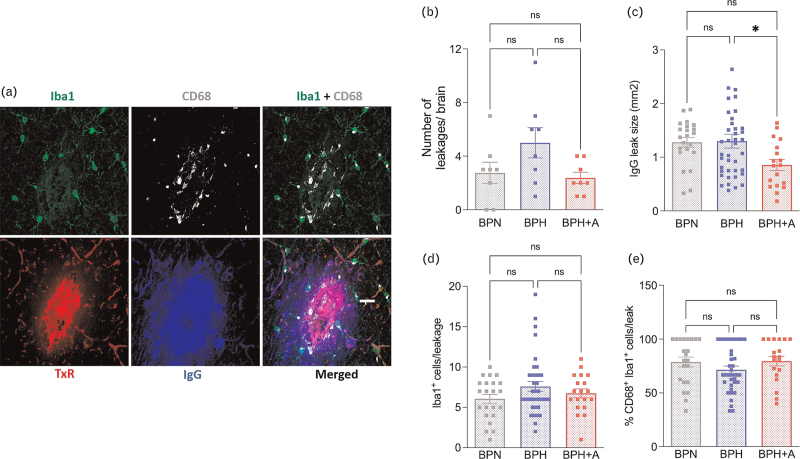
Amlodipine does not prevent blood–brain barrier leakages but limits their size. (a) Representative pictures of a blood–brain barrier (BBB) leakage identified in a BPH mouse by 70-kDa dextran-positive (red) and IgG-positive (blue) signals, Iba1^+^ cells (green) and CD68^+^ cells (grey) (scale bar = 50 μm). (b) The number of BBB leakages did not differ per group (1-W ANOVA *P* = 0.0782; *n* = 8 per group). (c) The average size of IgG leakages was smaller in BPH+A vs. BPH (1-W ANOVA *P* = 0.0496; Tukey's multiple comparison test: ^∗^*P* < 0.05 vs. BPH). (d) The average number of Iba1^+^ microglia per leakage did not differ between groups (1-W ANOVA *P* = 0.190). (e) The percentage of CD68^+^Iba1^+^ activated microglia per leakage did not differ between groups (1-W ANOVA *P* = 0.282).

BBB permeability was assessed by the extent of IgG extravasation into the brain parenchyma (Fig. 3a). Although the total number of leakages did not differ between the groups, the average leak size was smaller in BPH+A than in BPH (Fig. 3b, c). We observed no difference in microglia density (Iba1^+^ cells) and microglia activation (Iba1^+^CD68^+^ cells) at BBB leakage sites (Fig. 3a, d, e).

### Amlodipine and microglia's phenotype

**FIGURE 4:**
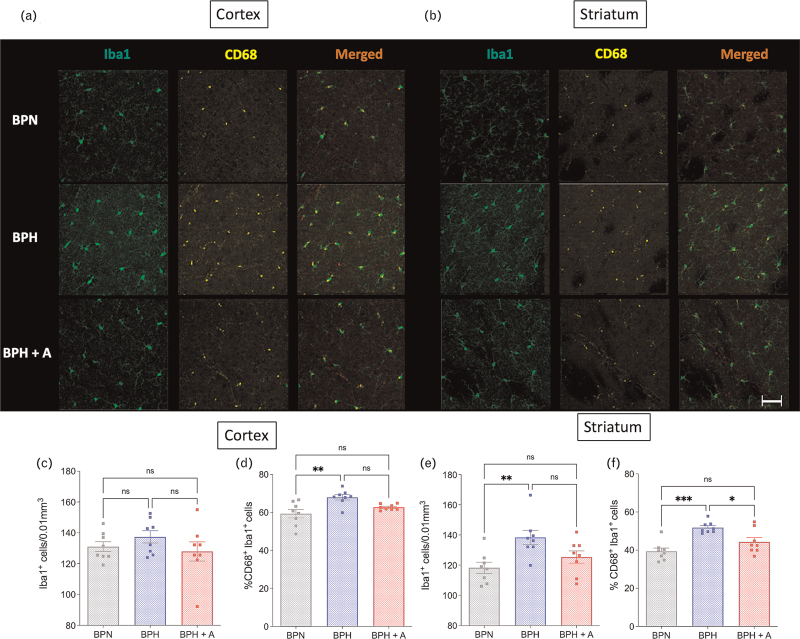
Microglia density and pro-inflammatory phenotype. Representative pictures of Iba1^+^ cells (green) and CD68^+^ cells (yellow) in the cortex (a) and striatum (b), (scale bar = 50 μm). (c) Iba1^+^ microglia densities in the cortex (1-W ANOVA *P* = 0.367; *n* = 8 per group). (d) Percentage of CD68^+^Iba1^+^ microglia in the cortex (1-W ANOVA *P* = 0.003; *n* = 8 per group). (e) Iba1^+^ microglia densities in the striatum (1-W ANOVA *P* = 0.009; *n* = 8 per group). (f) Percentage of CD68^+^Iba1^+^ microglia in the striatum (1-W ANOVA *P* < 0.001; *n* = 8 per group). Tukey's multiple comparison test (ns *P* > 0.05; ^∗^*P* < 0.05; ^∗∗^*P* < 0.01; ^∗∗∗^*P* < 0.001).

Microglia density (Iba1^+^ cells) and microglia activation (Iba1^+^ CD68^+^) were assessed in both the cortex (grey matterr) and striatum (white matter rich area) (Fig. 4a,b). In the cortex, the density of microglia did not differ between the groups (Fig. 4c). In BPH, there was a higher percentage of CD68^+^ microglia compared with BPN, although it did not differ in BPH+A compared with BPN mice (Fig. 4d). In the striatum, there was an increased microglia density observed in the BPH group compared with BPN (Fig. 4d). Furthermore, the percentage of CD68^+^ microglia was increased in BPH compared with BPN, while it was not increased in BPH+A compared with BPN group (Fig. 4f).

**FIGURE 5:**
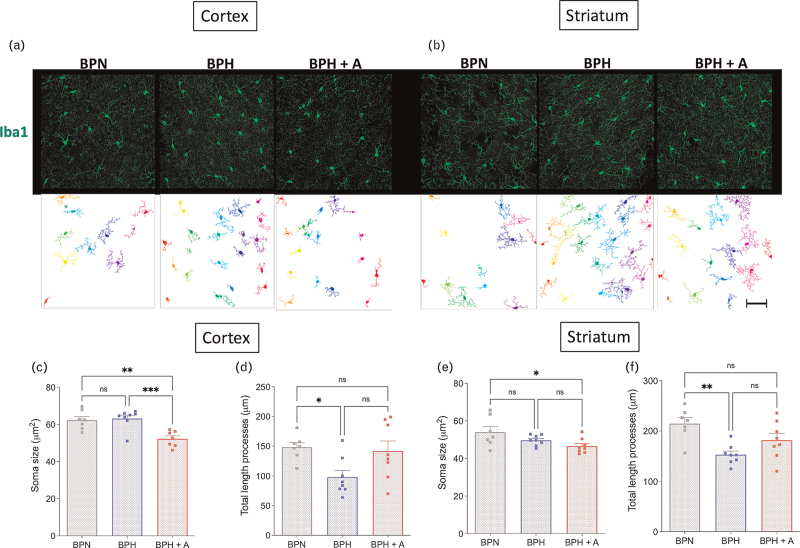
Microglia morphology. Representative images of Iba1^+^ cells (green) and their automatic morphological analyses using Neuromath for the cortex (a) and striatum (b) (scale bar = 50 μm). (c) Soma size of Iba1^+^ cells within the cortex (1-W ANOVA *P* < 0.001; *n* = 7–8 per group). (d) Length of the processes of the Iba1^+^ cells within the cortex (1-W ANOVA *P* = 0.023; *n* = 7–8 per group). (e) Soma size of Iba1^+^ cells within the striatum (1-W ANOVA *P* = 0.040; *n* = 7–8 per group). (f) Length of the processes of the Iba1^+^ cells within the striatum (1-W ANOVA *P* = 0.003; *n* = 7–8 per group). Tukey's multiple comparison test (ns *P* > 0.05; ^∗^*P* < 0.05; ^∗∗^*P* < 0.01; ^∗∗∗^*P* < 0.001).

We further assessed microglia's phenotype by performing a morphological analysis. In the cortex, the microglial soma size in BPH mice was larger than in BPH+A mice (Fig. 5c). In the striatum, we observed a smaller soma size in the BPH+A group compared with the BPN group but not different from the BPH group (Fig. 5e). The length of the microglia processes was shorter in the BPH group compared with the BPN group, although there was no difference between the BPN and BPH+A group for both the cortex and striatum (Fig. 5d,f).

## DISCUSSION

We aimed to study the ability of the CCB amlodipine to reduce BPv, alleviate BBB leakage and microglial activation and prevent cognitive dysfunction in aged hypertensive mice. Our study was able to confirm the lifelong increased blood pressure and blood pressure variability in BPH mice. We also found that BPH mice had worse cognitive performance as evidenced in the spatial short-term memory task as well as an altered spatial learning compared with BPN mice as shown in the Barnes Maze task. Although BBB dysfunction was not profound, BPH mice showed increased microglia activation compared with BPN mice.

A second important finding was that amlodipine treatment normalized the SBP of BPH mice for their entire lifespan. Furthermore, we showed that the BPv was also normal in these animals. Beyond its impact on blood pressure, amlopidine also attenuated the short-term memory decline observed in aged hypertensive mice. Interestingly, although amlodipine did not affect the number of BBB leaks in aged BPH mice, it did reduce the size of the leakages. A switch of micrcoglia towards a pro-inflammatorry phenotype is expected at the site of BBB leakages. Foci of BBB leaks were not showing any differences in microglia density and microglia activation between the study groups, suggesting that amlodipine is not sufficient to directly influence the behaviour of the microglia cells at the site of BBB leakages. We showed, however, that amlodipine was able to limit microglia's inflammatory phenotype in other brain regions.

In the present study, we used BPH mice that spontaneously develop hypertension, and exhibit increased BBB permeability with an impaired cognitive function [15,32]. We confirmed several traits of this animal model. Furthermore, we also verified the impact of amlodipine on BPv in these mice. Our results are in line with recent preclinical and clinical studies showing the benefit of the CCB amlodipine to reduce BPv and reduce target organ damage compared with other antihypertensive drug classes [20,21,33]. Beyond its impact on blood pressure, short-term memory impairment was also prevented by amlodipine in aged hypertensive mice. In spontaneous hypertensive rats, the CCB nimodipine could only partially reverse the learning and memory impairment induced by hypertension and ageing [34].

Due to its blood-pressure-lowering effect, we were expecting amlodipine to also decrease the number of BBB leaks. Although we have not observed a decrease in the number of BBB leaks, we found that amlodipine decreased the extent of the BBB damage. Both preclinical and clinical studies have shown the important association of an increased BBB permeability with cognitive dysfunction [35–37]. It is thought that changes in BBB integrity led to chronic change in neuro-inflammation status thereby disturbing normal brain homeostasis and consequently leading to cognitive dysfunction. In a previous study, we have shown that depletion of microglia and perivascular macrophages can prevent a short-term memory impairment in an Angiotensin II induced hypertensive mouse model [38]. Microglia are the brain resident immune cells and play a critical role in keeping brain homeostasis by regulating neuronal development and innate immune response [39]. Disruption of the BBB might cause the entrance of plasma component in the brain parenchyma with microglia activation as a consequence [16]. We observerd that the activation of microglia cells is increased surrounding BBB leakages (Fig. 3e) compared with the microglia cells in the other brain regions (Fig. 4d,f). Within BBB leaks, there was no difference in microglia density and microglia activation between the study groups, suggesting that amlodipine is not sufficient to directly influence the behaviour of the microglia cells at the site of BBB leakages. We showed however that amlodipine was able to limit microglia activation in other brain regions. CCBs have been shown to dampen the pro-inflammatory phenotype of microglia in vitro [26] but this had not been studied in vivo before. It remains however unknown if the prevention of microglia activation is solely due to a diminished BBB permeability, due to the SBP and BPv normalization, or rather caused by a direct impact of amlodipine on the signaling leading to microglial activation.

Although amlodipine does not cross the BBB acutely, it is possible that lipophilic dihydropyridines, including amlodipine, are able to cross the BBB with chronic treatments [40,41]. As intracellular calcium is important for the transition of microglia from their resting state to an activated state [42], the CCB amlodipine could therefore act directly on microglial activation. In fact, activation of microglia induced by lipopolysaccharide (LPS) results in an increased calcium influx, where blocking of calcium influx was able to reduce the release of pro-inflammatory cytokines [43]. These results suggests that the entry of extracellular calcium is essential for LPS induced microglia activation. Furthermore, it has been shown in vitro that CCB's have an anti-inflammatory effect by inhibiting microglia migration, activation and reduced expression of inflammatory markers [26]. These findings suggest neuroprotetive effects of CCB antihypertensive drugs beyond their blood pressure lowering effects. Targeting microglia calcium channels to prevent or improve central nervous system disorders seems to be a promising step for future studies [42].

Next to differential effects of antihypertensive drugs on neuroinflammation, their ability to prevent and/or restore cerebral blood flow reactivity may also differ, which is another important point of attention to prevent vascular dementia. Interestingly, in the study of Koide *et al.*[44], neurovascular coupling was found to be altered in BPH mice compared with BPN mice and this could be restored by amlodipine but not losartan treatment despite their equipotent blood pressure lowering effects [45]. The authors demonstrated that losartan was unable to restore neurovascular coupling due to the effects mediated by the aldosterone increase subsequent to the AT1R blockade. Taken together, this indicates that the protection afforded by amlodipine treatment against hypertension-induced cognitive decline in our study could also be mediated by restoration/improvement of neurovascular coupling. Indeed, if blood flow cannot be adjusted to match the neuronal activity, this may lead to reduced neuronal activity and delayed cognitive processes such as those observed in our study.

In summary, we have shown that amlodipine treatment was able to limit microglial inflammatory phenotype and short-term memory impairment associated with hypertension in aged mice. It remains unknown if this mechanism is independent of the effect of amlodipine on blood pressure, blood pressure variability nor neurovascular coupling. Furthermore, although it supports the use of CCB as blood pressure lowering strategy in hypertensive patients at risk for cerebrovascular lesions, it would be valuable to assess the impact of other antihypertensive drug classes on neuroinflammation.

## ACKNOWLEDGEMENTS

This project has received funding from the European Union's Horizon 2020 research and innovation programme under grant agreement No. 666881, SVDs@target.

### Conflicts of interest

There are no conflicts of interest.
